# Dexamethasone promotes renal fibrosis by upregulating ILT4 expression in myeloid‐derived suppressor cells

**DOI:** 10.1111/jcmm.18310

**Published:** 2024-04-27

**Authors:** Xiaowen Gu, Lianmei Zhang, Min Sun, Ying Zhou, Jinling Ji, YunFang Xu, Jianguo You, Zhikui Deng

**Affiliations:** ^1^ Department of Blood Transfusion The Affiliated Huaian No.1 People's Hospital of Nanjing Medical University Huai'an China; ^2^ Department of Science and Education Huai'an Municipal Center for Disease Control and Prevention Huai'an China; ^3^ Clinical Laboratory Huai'an No 4 People's Hospital Huai'an China

**Keywords:** dexamethasone, fibrosis, FSGS, ILT4, MDSCs

## Abstract

Studies have shown that adoptive transfer of myeloid‐derived suppressor cells (MDSCs) can alleviate various inflammatory diseases, including glomerulonephritis, but the long‐term effects of the transferred MDSCs are still unclear. In addition, although glucocorticoids exert immunosuppressive effects on inflammatory diseases by inducing the expansion of MDSCs, the impact of glucocorticoids on the immunosuppressive function of MDSCs and their molecular mechanisms are unclear. In this study, we found that adoptive transfer of MDSCs to doxorubicin‐induced focal segmental glomerulosclerosis (FSGS) mice for eight consecutive weeks led to an increase in serum creatinine and proteinuria and aggravation of renal interstitial fibrosis. Similarly, 8 weeks of high‐dose dexamethasone administration exacerbated renal interstitial injury and interstitial fibrosis in doxorubicin‐induced mice, manifested as an increase in serum creatinine and proteinuria, collagen deposition and α‐SMA expression. On this basis, we found that dexamethasone could enhance MDSC expression and secretion of the fibrosis‐related cytokines TGF‐β and IL‐10. Mechanistically, we revealed that dexamethasone promotes the expression of immunoglobulin‐like transcription factor 4 (ILT4), which enhances the T‐cell inhibitory function of MDSCs and promotes the activation of STAT6, thereby strengthening the expression and secretion of TGF‐β and IL‐10. Knocking down ILT4 alleviated renal fibrosis caused by adoptive transfer of MDSCs. Therefore, our findings demonstrate that the role and mechanism of dexamethasone mediate the expression and secretion of TGF‐β and IL‐10 in MDSCs by promoting the expression of ILT4, thereby leading to renal fibrosis.

## 1 INTRODUCTION

MDSCs were initially described in cancer patients^1^ and tumour‐bearing mice,^2^ represent a heterogeneous population of immature immunosuppressive cells, including myeloid progenitor cells and immature myeloid cells, and are widely involved in cancer, inflammation, infection and autoimmune disease.^3^ MDSCs that are expanded and activated under various pathological conditions negatively regulate the immune response by suppressing T‐cell proliferation, interfering with T‐cell trafficking and viability and inducing regulatory T (Treg) cells,^4^ promoting the release of cytokines such as IL‐10 and TGF‐β.^5^, ^6^ Based on morphological heterogeneity, MDSCs can be further divided into two subsets: monocytic MDSCs (M‐MDSCs) and granulocytic MDSCs (G‐MDSCs).^7^ M‐MDSCs and G‐MDSCs differ in their mechanisms underlying immunosuppressive function. G‐MDSCs generate high levels of reactive oxygen species (ROS) and low levels of nitric oxide (NO), while M‐MDSCs generate high levels of NO and low levels of ROS, and both subsets express Arginase 1.^8^ However, the current application of these molecular markers cannot distinguish immunosuppressive MDSCs with high specificity from nearby noninhibitory cells, which limits basic and clinical research on MDSCs.

Unlike in tumours, MDSCs can alleviate the progression of some inflammatory diseases, including inflammatory bowel disease,^9^, ^10^, ^11^, ^12^ rheumatoid arthritis,^13^, ^14^, ^15^, ^16^ autoimmune liver disease^17^, ^18^, ^19^ and glomerulonephritis.^20^, ^21^, ^22^, ^23^ In early studies, MDSCs demonstrated protective effects on asthma and airway inflammation by inhibiting TH2‐type immune responses.^24^, ^25^ Adoptive transfer of MDSCs to mice with allergic airway inflammation can reduce the severity of the disease. MDSCs also showed protective effects in mouse models of Sjogren's syndrome^26^ and arthritis.^27^ MDSCs are also associated with the occurrence or progression of inflammatory bowel disease.^9^ In a mouse model of colitis, the use of histone methyltransferase inhibitors alleviated disease progression by inducing the accumulation of immunosuppressive MDSCs in the colon.^28^ In addition, mTOR inhibitors and garamycin acetate have shown efficacy in mouse colitis by enhancing the inhibitory function of MDSCs.^29^ Li et al. demonstrated that glucocorticoids could induce MDSC expansion *in vivo* ^30^ and in vitro,^31^ and the change in MDSCs in focal segmental glomerulosclerosis (FSGS) patients to some extent reflected the efficacy of dexamethasone treatment. Cortisol and synthetic glucocorticoid analogues activate intracellular glucocorticoid receptors (GRs) and regulate many physiological processes. Due to the powerful effects of glucocorticoids on immune cells, they have long been the most widely prescribed anti‐inflammatory and immunosuppressive drugs.^32^ However, patients treated with glucocorticoids may exhibit resistance or widespread adverse effects, such as high‐dose pulse therapy leading to pulmonary fibrosis,^33^ and the molecular mechanism has not yet been fully elucidated.

Immunoglobulin‐like transcription factor 4 (ILT4), also known as leukocyte immunoglobulin‐like receptor B2 (LILRB2), consists of four extracellular Ig domains, one transmembrane domain and three cytoplasmic ITIM motifs and is mainly expressed in myeloid cells, including monocytes, macrophages, granulocytes and dendritic cells (DCs).^34^, ^35^ ILT4 is highly expressed in various solid tumours, including non‐small cell lung cancer (NSCLC), breast cancer, oesophageal cancer and pancreatic cancer, to promote tumour proliferation and growth.^36^, ^37^, ^38^, ^39^, ^40^ At the same time, it is also expressed in stromal cells in the tumour suppression microenvironment to facilitate tumour progression and metastasis.^38^ The typical ligand of ILT4 is human leukocyte antigen G (HLA‐G), a nonclassical MHC class I molecule widely expressed in tumour cells, and its expression is associated with poor prognosis. Several other ILT4 ligands, including classic human leukocyte antigen (HLA) and angiopoietin‐like proteins, may also be associated with driving ILT4‐mediated tumour microenvironmental immunosuppression.^41^ In addition, ILT4 is also associated with various inflammatory diseases, such as rheumatoid arthritis and psoriatic arthritis.^42^, ^43^ Although there have been studies on the immunosuppressive function of surface ILT4 in inflammatory diseases, the molecular mechanism of its expression regulation is unclear.

In this study, we found that long‐term adoptive transfer of MDSCs aggravates renal fibrosis, and long‐term high‐dose administration of dexamethasone leads to a similar consequence. A mechanical study revealed that dexamethasone increases the expression of ILT4 on MDSCs, which promotes the activation of STAT6, thereby enhancing the expression and secretion of TGF‐β and IL‐10.

## 2 RESULTS

### 2.1 Long‐term adoptive transfer of MDSCs promotes renal fibrosis

**FIGURE 1:**
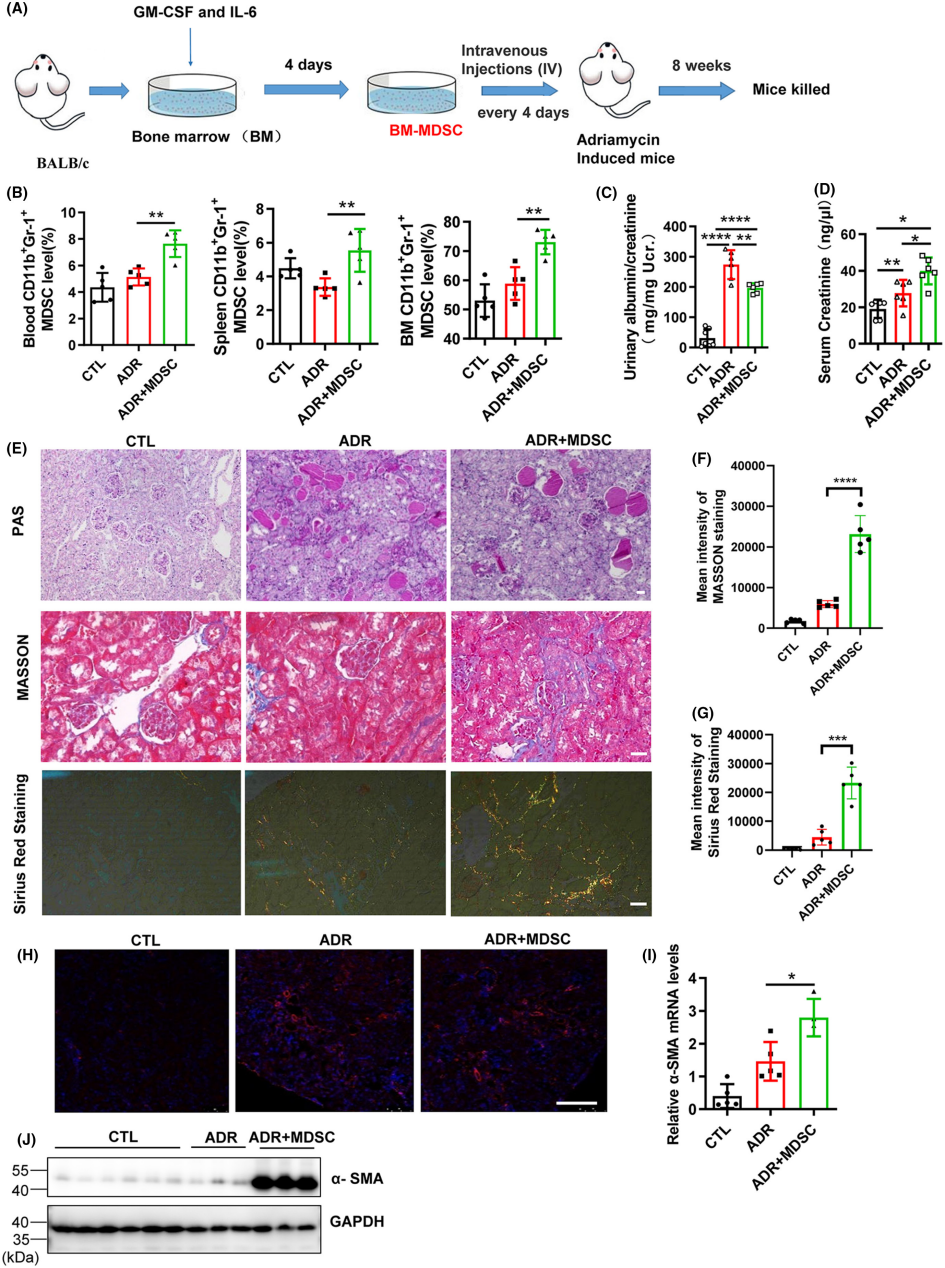
Long‐term administration of MDSCs promotes renal fibrosis. (A) Diagram of the experimental design. BM‐derived MDSCs were prepared and injected intraperitoneally into mice 1 day after ADR injection and injected every 4 days for 8 weeks. Mice were grouped as follows: 0.9% NaCl (CTL, *n* = 5), adriamycin (ADR, *n* = 5), ADR plus 5 × 10^6^ induced BM‐MDSCs (ADR + MDSC, *n* = 5). After 8 weeks, the mice were euthanized and subjected to renal pathology and biochemical analysis. (B) Flow cytometry analysis of MDSCs in peripheral blood, spleen and bone marrow. (C) Urinary albumin/creatinine ratios. (D) Serum creatinine levels. (E) Representative PAS‐, Masson‐ and Sirius Red‐stained renal sections. Scale bar, 100 μm. (F, G) Statistical analysis of Masson and Sirius Red staining results. (H) Representative immunofluorescence staining of a‐SMA. Scale bar, 100 μm. (I) The mRNA level of a‐SMA was determined by RT–qPCR. (J) Protein levels of a‐SMA were determined by western blotting. *p < 0.05; **p < 0.01; ***p < 0.001 and ****p < 0.0001.

To investigate the long‐term therapeutic effect of adoptive transfer of MDSCs, we first induced bone marrow (BM) from BALB/c mice to obtain MDSCs using the cytokines GM‐CSF and IL‐6 in vitro and then injected induced BM‐derived MDSCs (BM‐MDSCs, 5 × 10^6^ cells per mouse) every 4 days into adriamycin (ADR)‐induced BALB/c mice through the tail vein, and the mice were euthanized 8 weeks later to investigate the effects of adoptive transfer of MDSCs on biochemical and pathological indicators in ADR‐induced mice (Figure 1A). Previous studies have shown that short‐term adoptive transfer of MDSCs can alleviate podocyte damage and proteinuria in ADR‐induced mice. Our results show that adoptive transfer of BM‐MDSCs could significantly increase the MDSC content in ADR‐induced mice (Figure 1B), and long‐term adoptive transfer of BM‐MDSCs can alleviate proteinuria to some extent (Figure 1C), but it exacerbates renal tubular injury and interstitial fibrosis, manifested by increased serum creatinine levels (Figure 1D) and increased collagen deposition in the tubulointerstitium (Figure 1E–G), respectively. In addition, examination of α‐SMA protein through immunofluorescence showed an increase in its expression, mainly in renal tubules (Figure 1H). Further isolation of renal tubules from mouse kidneys confirmed the significant increase in α‐SMA protein and mRNA expression in renal tubules (Figure 1I,J), confirming the role of long‐term adoptive transfer of MDSCs in promoting renal fibrosis.

### 2.2 Long‐term administration of high‐dose dexamethasone promotes renal fibrosis

**FIGURE 2:**
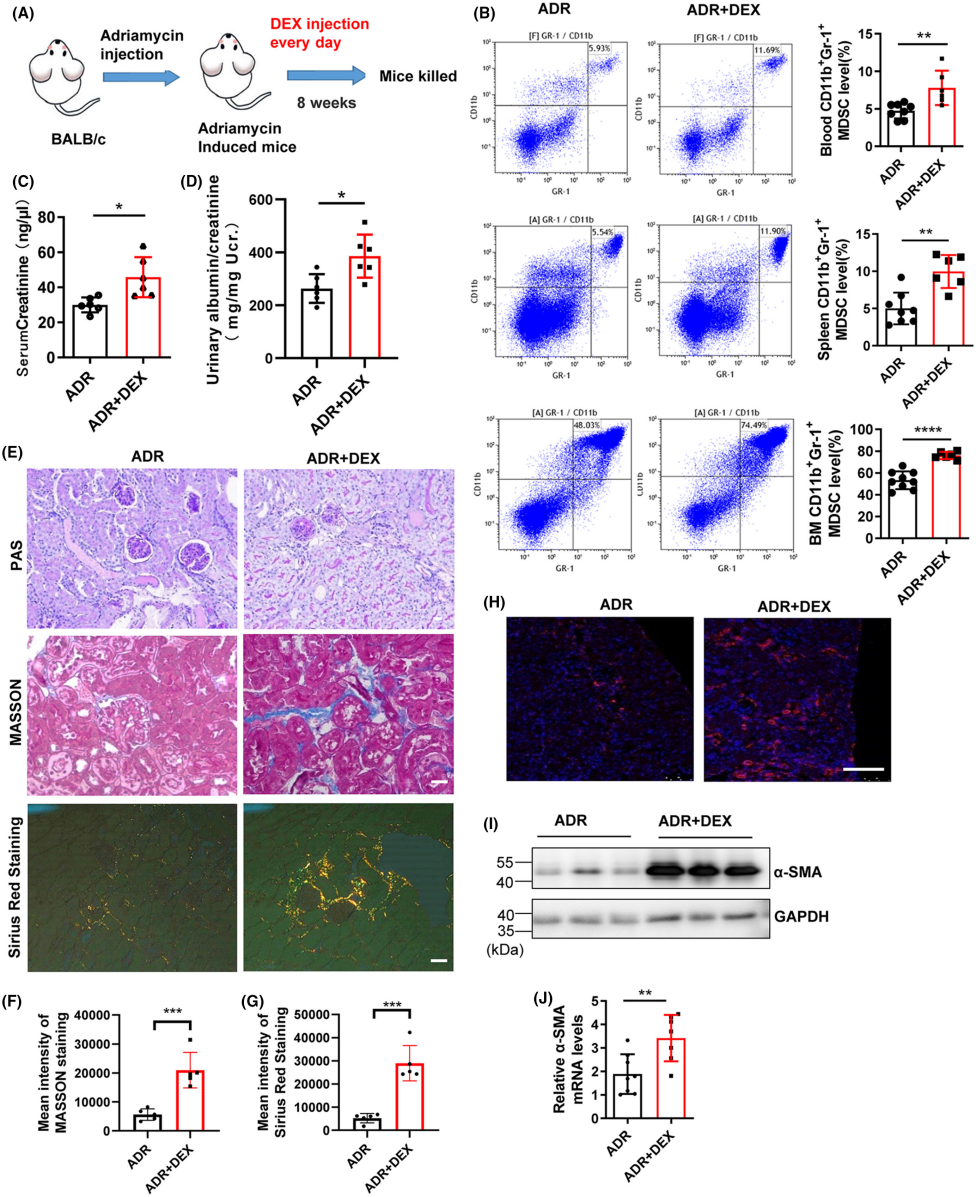
Long‐term administration of high‐dose dexamethasone promotes renal fibrosis. (A) Diagram of the experimental design. Dexamethasone (DEX, 10 mg/kg) was injected intraperitoneally into mice 1 day after ADR injection and injected every day for 8 weeks. Mice were grouped as follows: adriamycin (ADR, *n* = 5) and ADR plus 1 mg/kg dexamethasone (ADR + DEX, *n* = 5). After 8 weeks, the mice were euthanized and subjected to renal pathology and biochemical analysis. (B) Flow cytometry analysis of MDSCs in peripheral blood, spleen and bone marrow. (C) Serum creatinine levels. (D) Urinary albumin/creatinine ratios. (E) Representative PAS‐, Masson‐ and Sirius Red‐stained renal sections. Scale bar, 100 μm. (F and G) Statistical analysis of Masson and Sirius Red staining results. (H) Representative immunofluorescence staining of a‐SMA. Scale bar, 100 μm. (I) Protein levels of a‐SMA were determined by western blotting. (J) The mRNA level of a‐SMA was determined by RT–qPCR. *p < 0.05; **p < 0.01; ***p < 0.001 and ****p < 0.0001.

Studies have shown that short‐term low‐dose dexamethasone, an immunosuppressive reagent widely used to treat focal segmental glomerular sclerosis (FSGS) patients,^44^ could induce MDSC expansion and suppress T‐cell‐mediated inflammation in FSGS patients.^30^ We thus tested whether long‐term high‐dose dexamethasone administration has a similar effect as adoptive transfer of MDSCs. We administered adriamycin to mice with dexamethasone (DEX) at a dose of 10 mg/kg per day for eight consecutive weeks, and the mice were euthanized to examine the biochemical and pathological indicators (Figure 2A). We found that high‐dose dexamethasone treatment for eight consecutive weeks significantly increased the MDSC levels in ADR‐induced mice (Figure 2B), whereas it failed to alleviate renal injury and significantly increased serum creatinine (Figure 2C) and proteinuria (Figure 2D) levels and aggravated interstitial fibrosis (Figure 2E–G) compared to those without dexamethasone administration, suggesting that long‐term high‐dose dexamethasone administration promotes renal tubular injury and interstitial fibrosis. Immunofluorescence detection of the fibrotic marker protein α‐SMA showed an increase in its expression in renal tubules (Figure 2H). Further isolation of renal tubules from mouse kidneys confirmed the significant increase in α‐SMA protein (Figure 2I) and mRNA (Figure 2J) expression in renal tubules, confirming the role of long‐term high‐dose dexamethasone administration in promoting renal fibrosis.

### 2.3 Dexamethasone promotes MDSC secretion of TGF‐β and IL‐10

**FIGURE 3:**
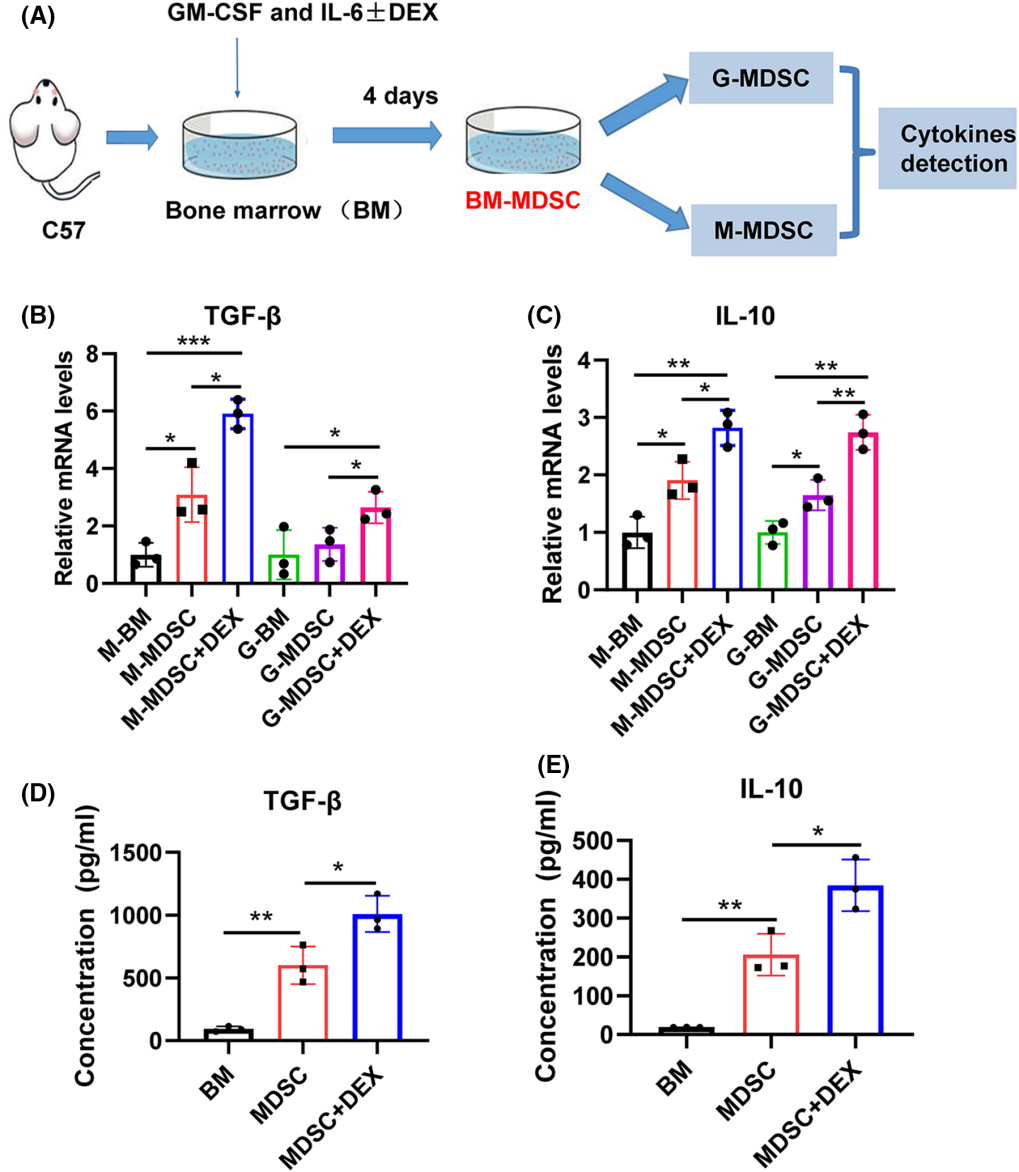
Dexamethasone promotes MDSC expression and secretion of TGF‐β and IL‐10. (A) Bone marrow cells from BALB/c mice were induced by GM‐CSF and IL‐6 with or without dexamethasone (DEX). Cells were grouped as follows: BM without cytokines and dexamethasone treatment (BM), BM with GM‐CSF and IL‐6 treatment (MDSC), BM with GM‐CSF, IL‐6 and dexamethasone treatment (MDSC + DEX). Monocytic and granulocytic cells were sorted from the BM (M‐BM, G‐BM), MDSC (M‐MDSC, G‐MDSC) and MDSC + DEX (M‐MDSC + DEX, G‐MDSC + DEX) groups. TGF‐β1 and IL‐10 levels were determined. (B) TGF‐β1 mRNA levels were measured by qRT–PCR. (C) IL‐10 mRNA levels were measured by qRT–PCR. (D) The concentration of TGF‐β1 in cell culture supernatants was determined by ELISA. (E) The concentration of IL‐10 in cell culture supernatants was determined by ELISA. *p < 0.05; **p < 0.01; ***p < 0.001.

Numerous studies have confirmed the profibrotic effect of TGF‐β and IL‐10.^45^, ^46^ It is known that IL‐10 can reduce the expression of IL‐2, INF‐γ, TNF‐α and GM‐CSF, which have anti‐inflammatory effects, thereby reducing the inflammatory response in the early stage of fibrosis and promoting the progression of fibrosis in the later stage.^47^ TGF‐β can regulate cell growth, differentiation and extracellular matrix synthesis and plays an important role in development, immune and inflammatory processes.^48^ Therefore, we reasonably speculate that long‐term adoptive transfer of MDSCs and high‐dose dexamethasone treatment promote renal fibrosis through TGF‐β and IL‐10 secretion. Therefore, we detected the expression of TGF‐β and IL‐10 in MDSCs as well as the effect of dexamethasone on them. We induced BM‐MDSCs using GM‐CSF and IL‐6 while also administering dexamethasone or not. After inducing BM‐MDSCs, we sorted M‐MDSC and G‐MDSC and detected TGF‐β and IL‐10 expression in both cell populations (Figure 3A). Compared to that in BM without cytokine induction (M‐BM), the expression of TGF‐β in GM‐CSF‐ and IL‐6‐induced M‐MDSCs was significantly increased, and treatment with dexamethasone further increased TGF‐β in M‐MDSCs (Figure 3B). Compared with BM without cytokine induction (G‐BM), there was no significant increase in the expression of TGF‐β in G‐MDSCs, but after dexamethasone treatment, TGF‐β expression in G‐MDSCs increased significantly (G‐MDSC+DEX, Figure 3B). The expression of IL‐10 in MDSCs was similar to that of TGF‐β, except that IL‐10 expression in G‐MDSCs was significantly increased compared to that in BM without cytokine induction (G‐BM, Figure 3C). On this basis, we further tested the secretion of TGF‐β and IL‐10 in cell culture supernatants and found that, compared with BM culture supernatant without cytokine induction, both TGF‐β and IL‐10 secretion in MDSC culture supernatant with cytokine induction increased significantly (Figure 3D,E). Moreover, dexamethasone treatment further enhanced TGF‐β and IL‐10 secretion in the MDSC culture supernatant (Figure 3D,E). These results indicate that GM‐CSF and IL‐6 induce the expression and secretion of TGF‐β and IL‐10 in BM‐MDSCs themselves, and dexamethasone further promotes the expression and secretion of TGF‐β and IL‐10 in MDSCs.

### 2.4 Dexamethasone enhances the T‐cell inhibitory function of MDSCs by promoting ILT‐4 expression

**FIGURE 4:**
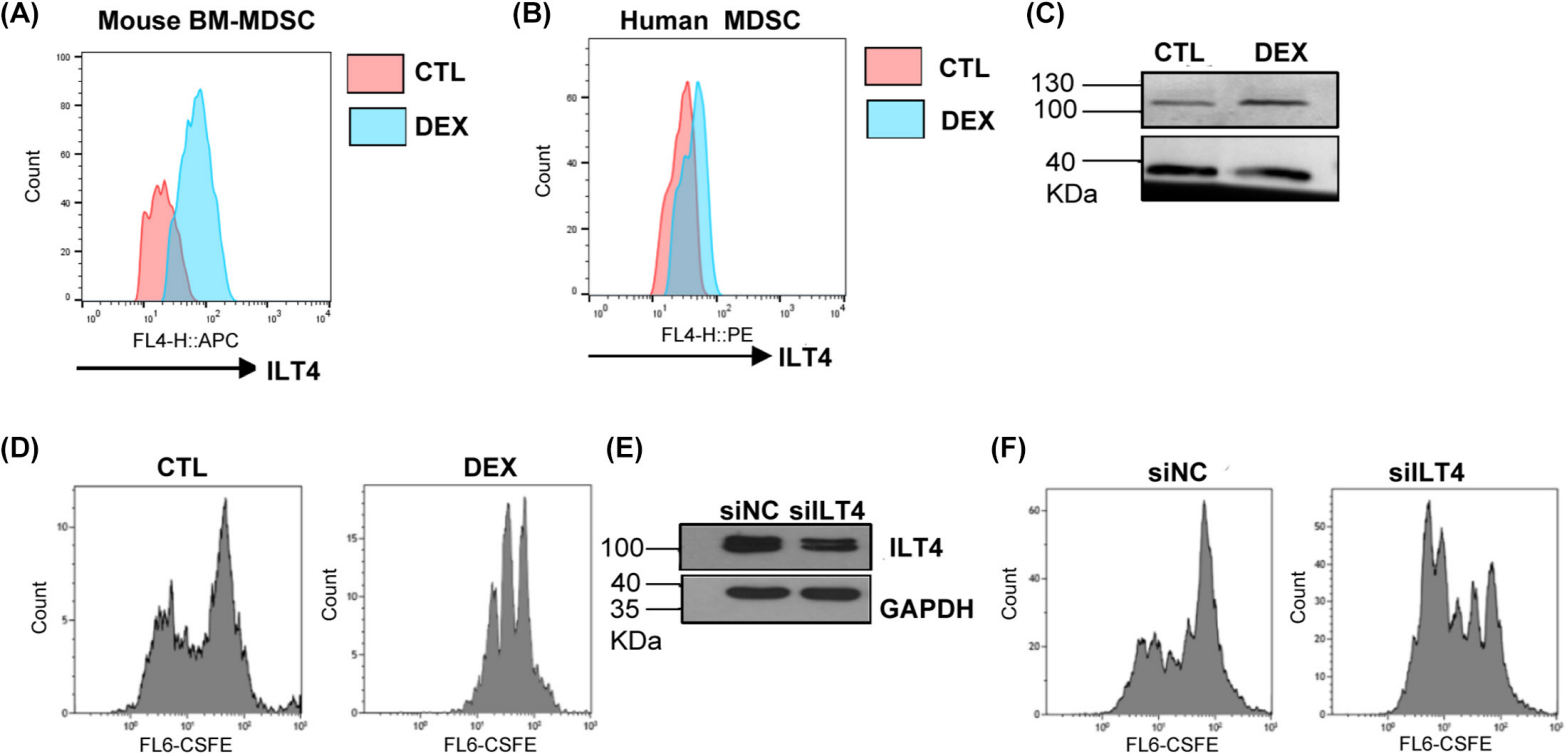
Dexamethasone enhances the T‐cell inhibitory function of MDSCs by promoting ILT4 expression. (A) Flow cytometry analysis of the expression of ILT4 on mouse CD11b^+^Gr‐1^+^ BM‐MDSCs under treatment with dexamethasone (DEX) or not (CTL). (B) Flow cytometry analysis of the expression of ILT4 on CD11b^+^CD33^+^HLA‐DR^low/−^ MDSCs from the peripheral blood of FSGS patients receiving glucocorticoid therapy (DEX) or not receiving glucocorticoid therapy (CTL). (C) western blotting analysis of the expression of ILT4 in BM‐MDSCs with or without dexamethasone treatment. (D) Comparison of the T‐cell suppressive ability between BM‐MDSCs with or without dexamethasone treatment. (E) Knocking down ILT4 in BM‐MDSCs using ILT4 siRNA (siILT4). Random siRNA sequences were used as controls (siNC). The protein levels were measured. (F) Comparison of the T‐cell suppressive ability between BM‐MDSCs with or without ILT4 knockdown.

ILT4 is mainly expressed in immune cells and can recruit the phosphatases SHP‐1, SHP‐2 or SHIP to negatively regulate cell activation and immune responses and inhibit adult neuronal cell regeneration.^49^ Previous studies have shown that MDSCs express ILT4,^50^ but the direct impact of ILT4 on MDSC immunosuppressive function as well as its anti‐inflammatory effects and molecular mechanisms, especially in renal inflammation and fibrosis, have not been fully elucidated. We found through flow cytometry analysis that dexamethasone induced ILT‐4 expression in GM‐CSF‐ and IL‐6‐induced BM‐MDSCs (Figure 4A). Consistent with this, FSGS patients treated with glucocorticoids for 7 days also showed an increase in the expression of ILT4 in peripheral MDSCs (Figure 4B). Western blotting results also showed the induction effect of dexamethasone on ILT4 expression in BM‐MDSCs (Figure 4C). On this basis, we further analysed the effect of ILT4 on the T‐cell inhibitory function of BM‐MDSCs through T‐cell proliferation experiments. First, we confirmed that dexamethasone treatment can enhance the inhibitory effect of BM‐MDSCs on T‐cell proliferation (Figure 4D). Then, we used small RNA interference technology to interfere with the expression of ITL4 during BM‐MDSC induction and found that reducing ILT4 expression with siRNA significantly reduced the inhibitory effect of BM‐MDSCs on T‐cell proliferation (Figure 4E,F). These results suggest that dexamethasone may enhance the T‐cell inhibitory effect of BM‐MDSCs by inducing the expression of ILT‐4.

### 2.5 ILT4 mediates the secretion of TGF‐β and IL‐10 in MDSCs by activating STAT6

**FIGURE 5:**
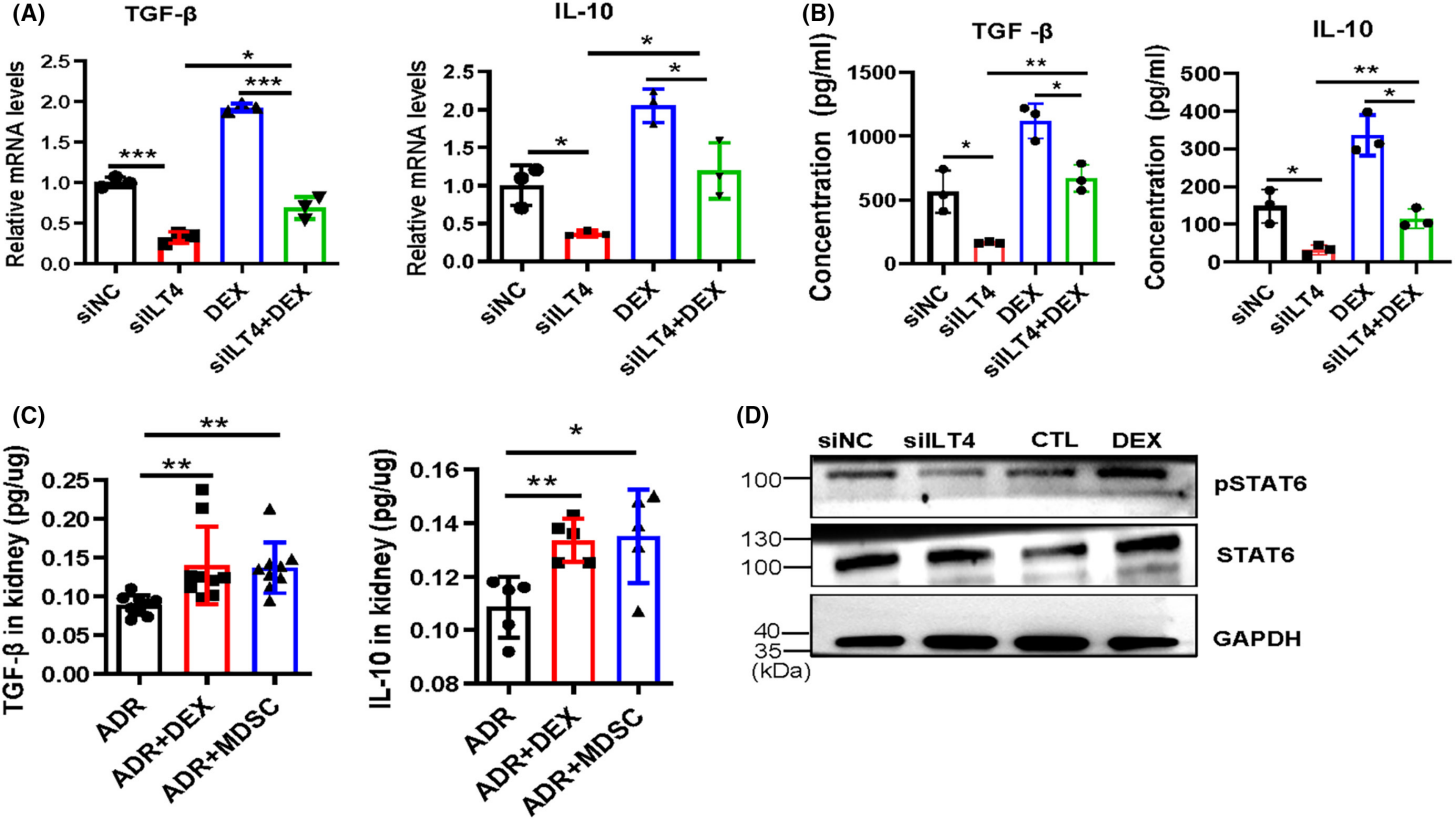
ILT4 mediates the expression and secretion of TGF‐β and IL‐10 in MDSCs by activating STAT6. (A) Bone marrow cells from BALB/c mice were induced by GM‐CSF and IL‐6 with or without dexamethasone (DEX). Simultaneously, ILT4 was knocked down using ILT4 siRNA (siILT4). Cells were grouped as follows: GM‐CSF and IL‐6‐induced BM transfected with random siRNA sequences (siNC), GM‐CSF and IL‐6‐induced BM transfected with ILT4 siRNA (siILT4), GM‐CSF and IL‐6‐induced BM plus dexamethasone treatment (DEX), and GM‐CSF and IL‐6‐induced BM transfected with ILT4 siRNA plus dexamethasone treatment (siILT4 + DEX). TGF‐β1 and IL‐10 levels were determined by qRT–PCR. (B) Concentrations of TGF‐β1 and IL‐10 in cell culture supernatants were determined by ELISA. (C) Concentrations of TGF‐β1 and IL‐10 in mouse kidney homogenates were determined and normalized to kidney weight. (D) western blotting analysis of the expression of STAT6 and pSTAT6 in different cell groups. *p < 0.05; **p < 0.01; ***p < 0.001.

Studies have shown that in the presence of M‐CSF and IL‐4, the antagonistic effect of specific monoclonal antibodies on ILT4 can inhibit the activation of AKT and STAT6, enhance the inflammatory response of monocytes, and directly alter the mature phenotype of downstream macrophages, suggesting the inhibitory effect of ILT4 on inflammation and macrophage phenotype differentiation.^51^ Since bone marrow‐derived MDSCs express ILT4 and dexamethasone further induces the expression of ILT4 in BM‐MDSCs, we next validated the effect of ILT4 on TGF‐β and IL‐10 expression and secretion in MDSCs. We observed that knocking down ILT4 inhibits the expression and secretion of IL‐10 and TGF‐β in MDSCs (Figure 5A,B), and knocking down ILT4 can resist dexamethasone‐induced TGF‐β and IL‐10 expression and secretion (Figure 5A,B). Similarly, comparative analysis of renal tissue homogenates from the ADR, ADR + DEX and ADR + MDSC groups of mice also revealed that dexamethasone and BM‐MDSC administration significantly enhanced the concentrations of TGF‐β and IL‐10 in renal tissues (Figure 5C). A mechanistic study found that ILT4 in MDSCs plays a role by promoting the activation of STAT6, manifested by knocking down ILT4 and inhibiting the expression of pSTAT6, while dexamethasone promotes the expression of pSTAT6 (Figure 5D).

### 2.6 Adoptive transfer of MDSCs with ILT4 knockdown alleviates renal fibrosis

**FIGURE 6:**
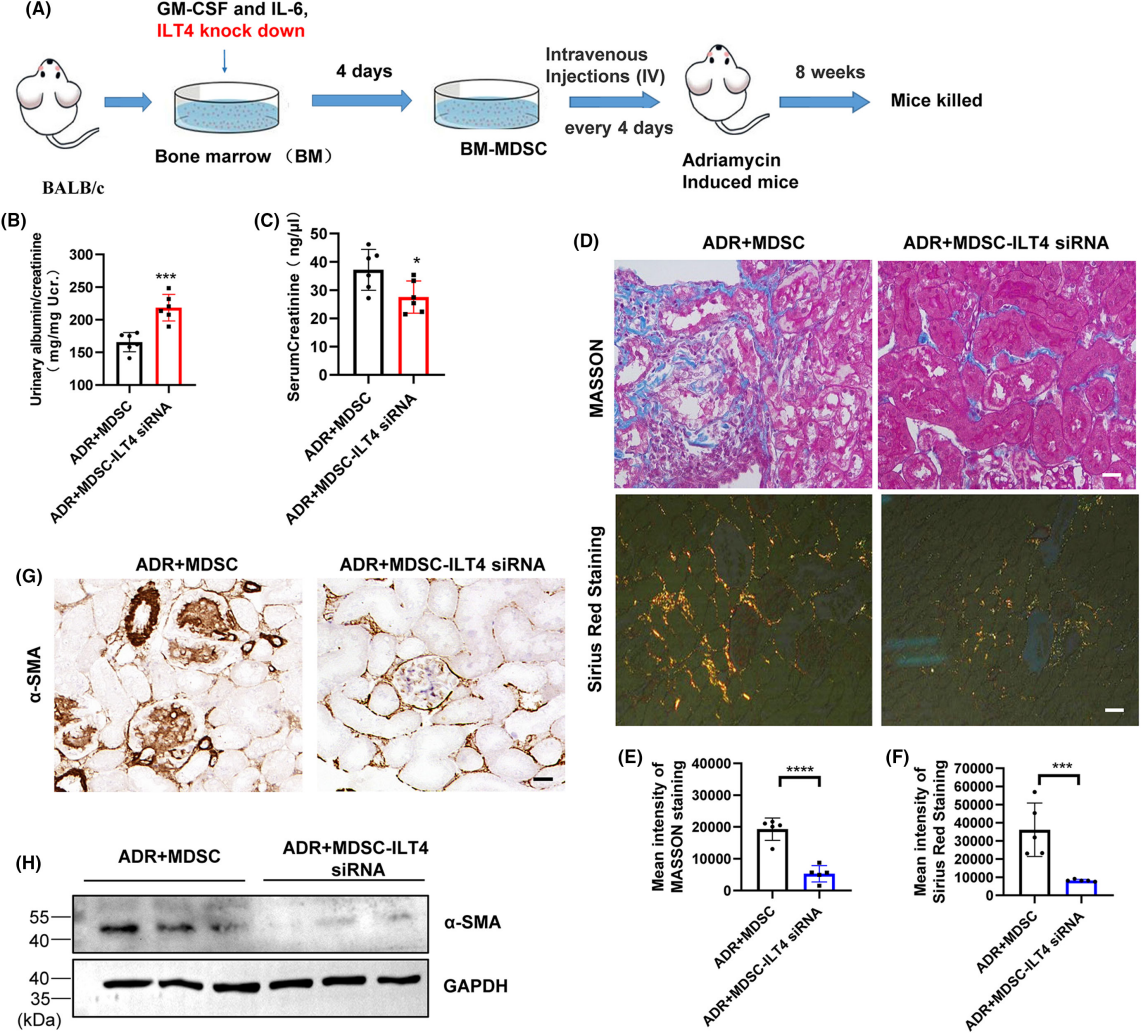
Adoptive transfer of MDSCs with ILT4 knockdown alleviates renal fibrosis. (A) Diagram of the experimental design. BM‐derived MDSCs transfected with ILT4 siRNA were prepared and injected intraperitoneally into mice 1 day after ADR injection and injected every 4 days for 8 weeks. Mice were grouped as follows: ADR plus 5 × 10^6^ induced BM‐MDSCs (ADR + MDSC, *n* = 5) and ADR plus 5 × 10^6^ induced BM‐MDSCs transfected with ILT4 siRNA (ADR + MDSC‐ILT4 siRNA, *n* = 5). After 8 weeks, the mice were euthanized and subjected to renal pathology and biochemical analysis. (B) Urinary albumin/creatinine ratios. (C) Serum creatinine levels. (D) Representative Masson and Sirius Red‐stained renal sections. Scale bar, 100 μm. (E, F) Statistical analysis of Masson and Sirius Red staining results. (G) Representative immunohistochemical staining of a‐SMA. Scale bar, 100 μm. (H) Protein levels of a‐SMA were determined by western blotting. *p < 0.05; ***p < 0.001; ****p < 0.0001.

To further clarify whether ILT4 mediates the fibrogenic effects of long‐term adoptive transfer of BM‐MDSCs in the kidney, we knocked down ILT4 with siRNA during the induction of BM‐MDSCs and then transferred them (5 × 10^6^ cells per mouse) to ADR‐induced BALB/c mice through the tail vein. Mice were grouped as follows: ADR plus 5 × 10^6^ BM‐MDSCs (ADR + MDSC, *n* = 5) and ADR plus 5 × 10^6^ BM‐MDSCs with ILT4 knockdown (ADR + MDSC‐ILT4 siRNA, *n* = 5). Mice were euthanized 8 weeks later for biochemical and pathological analysis (Figure 6A). Our results show that long‐term adoptive transfer of BM‐MDSCs with ILT4 knockdown significantly aggravated proteinuria (Figure 6B) but significantly alleviated renal tubular injury and interstitial fibrosis, as manifested by decreased serum creatinine levels (Figure 6C) and decreased collagen deposition in the tubulointerstitium (Figure 6D–F), suggesting that the transfer of MDSCs with ILT4 knockdown alleviates renal tubular injury and interstitial fibrosis. Immunohistochemical detection of the fibrotic marker protein α‐SMA showed that its expression was decreased in renal tubules (Figure 2G). Further isolation of renal tubules from mouse kidneys confirmed the significant increase in α‐SMA protein (Figure 2H) expression in renal tubules, confirming the role of ILT4 in MDSC‐mediated renal fibrosis.

## 3 DISCUSSION

The immune system is known to play a pivotal role in the development and progression of human tumours and inflammatory diseases.^52^ However, the precise mechanisms by which this protumorigenic or anti‐inflammatory activity occurs remain elusive. Clinical studies have shown that MDSCs are critical inflammatory suppressive cells correlated with good clinical outcomes,^53^ and adoptive transfer of MDSCs inhibits inflammation progression.^54^ Most previous studies have focused on the alleviating effect of adoptive transfer of MDSCs on inflammation, but the role of long‐term adoptive transfer of MDSCs within the microenvironment of human inflammatory diseases has yet to be well characterized. In this study, we demonstrated that long‐term adoptive transfer of MDSCs to mice induced by doxorubicin resulted in poor prognosis. We found that adoptive transfer of MDSCs to doxorubicin‐induced FSGS mice for 8 weeks led to an increase in serum creatinine and proteinuria and aggravation of renal interstitial fibrosis, manifested by increased collagen deposition in Masson staining results, increased expression of α‐SMA protein in immunofluorescence and western blotting results, and increased expression of α‐SMA and collagen I mRNAs in qRT–PCR results. More importantly, high‐dose dexamethasone also has similar effects as long‐term adoptive transfer of MDSCs on promoting renal fibrosis. Dexamethasone, as a member of the glucocorticoid family, has extensive immunosuppressive effects and has been reported to induce MDSC expansion both in vivo and in vitro.^30^, ^31^ Therefore, our results are consistent with the conclusion that high‐dose use of glucocorticoids has side effects in promoting pulmonary fibrosis.

The expression and biological role of costimulatory molecules, such as CD40, CD80 and DC‐sign, on MDSCs have attracted a great deal of attention in recent years.^55^ In this study, we found that ILT4 was expressed on GM‐CSF‐ and IL‐6‐induced BM‐MDSCs. In addition, knocking down ITL4 significantly reduces the T‐cell inhibitory ability of MDSCs in vitro and significantly reduces MDSC‐induced renal fibrosis in ADR‐induced mice in vivo, manifested by the decreased collagen deposition in Masson staining results and decreased expression of α‐SMA and collagen I proteins in immunohistochemical results. Although the exact immunological function of ITL4 in MDSCs within the inflammatory microenvironment remains to be defined, our current study demonstrates that ITL4 may serve as a marker expressed on MDSCs and reflect the unique immunomodulatory effects on renal fibrosis. We also attempted to determine what factor is important for ITL4 expression on MDSCs. We found that dexamethasone induces ITL4 expression in GM‐CSF‐ and IL‐6‐induced BM‐MDSCs in vitro. The expression of ILT4 on MDSCs from the peripheral blood of FSGS patients treated with glucocorticoids also increased significantly. Our data suggest that the immunosuppressive microenvironment is causal, inducing MDSCs to express ILT4. Other factors may also contribute to dexamethasone induction. For example, the costimulatory molecule CD40, a factor previously shown to be involved in reinforcing CD40 signalling, synergizes with the IL‐4/STAT6 pathway.^56^ Future studies are needed to further address this question in detail.

Numerous studies have confirmed that TGF‐β and IL‐10 are two key fibrosis‐related cytokines. IL‐10 can reduce the expression of IL‐2, INF‐γ, TNF‐α and GM‐CSF, which have anti‐inflammatory effects, thereby reducing the inflammatory response in the early stage of fibrosis and promoting the progression of fibrosis in the later stage.^47^ TGF‐β can regulate cell growth, differentiation and extracellular matrix synthesis.^45^ Of particular relevance, TGF‐β and IL‐10 have been previously shown to play a key role in MDSC‐mediated induction of Tregs.^57^ Consistent with this, our data showed that GM‐CSF‐ and IL‐6‐induced BM‐MDSCs also express and secrete TGF‐β and IL‐10. More importantly, we found that dexamethasone can further promote BM‐MDSC expression and secretion of TGF‐β and IL‐10, and this promoting effect is achieved by mediating the expression of ILT4. Knocking down ITL4 in MDSCs significantly reduces the expression and secretion of TGF‐β and IL‐10 and resists dexamethasone‐induced TGF‐β and IL‐10 expression and secretion. Studies have shown that specific monoclonal antibodies against ILT4 can inhibit the activation of AKT and STAT6, enhance the inflammatory response of monocytes, and directly alter the mature phenotype of downstream macrophages, suggesting the inhibitory effect of ILT4 on inflammation and macrophage phenotype differentiation.^51^ Similarly, our data demonstrated that ILT4 mediates the activation of STAT6 in bone marrow‐derived MDSCs. Knocking down ITL4 inhibits pSTAT6 expression, while dexamethasone promotes pSTAT6 expression. The limitation of our study is that we did not validate the function of ILT4‐knocked‐out MDSCs in vitro and in vivo. Using ILT4 knockout mice for validation will make our conclusions more convincing.

Based on the current findings, we propose a novel mechanism of adoptive transfer of MDSC‐mediated fibrosis. Adoptively transferred MDSCs secrete a number of factors, such as TGF‐β and IL‐10, via the ITL4 signalling pathway that stimulates STAT6 activation. In the complex environment of ADR‐induced mice, TGF‐β and IL‐10 are released by adoptively transferred MDSCs, activating fibrotic signalling pathways and promoting renal fibrosis. The fibrotic side effect of high‐dose glucocorticoids is also mediated by a similar mechanism by inducing ITL4 expression on MDSCs. Numerous studies have shown that MDSCs have strong immunosuppressive activity. Currently, different strategies are being used to regulate the function of MDSCs in tumours, including inhibiting their recruitment, accumulation and immunosuppressive function. In autoimmune diseases, it is to increase the accumulation of MDSCs or adoptive transfer of MDSCs. According to our findings, adoptive transfer of MDSCs in the treatment of inflammation or autoimmune diseases may also increase the risk of fibrosis. This is an issue that cannot be ignored. In future MDSC adoptive transfer therapy strategies, the number of cells and duration of treatment may need to be highly valued.

## 4 MATERIALS AND METHODS

### 4.1 Patients

The Affiliated Huai'an No.1 People's Hospital of Nanjing Medical University, Huai'an, China, approved all protocols concerning the use of patient samples in this study. Each donor provided a signed consent form. Blood samples were collected from consenting healthy donors and FSGS patients sensitive to glucocorticoids (GC) for ITL4 expression analysis of MDSCs. The patients were selected according to the following criteria: (1) proteinuria >0.4 g/24 h; eGFR ≥ 15 mL/min/per 1.73 m2; and (2) 18 < age < 65. Patients were excluded if they had obvious secondary factors, other glomerular diseases, tumours or a clear family history of kidney disease.

### 4.2 Animals

Animal maintenance and experimental procedures were carried out in accordance with the National Institute of Health Guidelines for Use of Experimental Animals and approved by the Affiliated Huai'an No.1 People's Hospital of Nanjing Medical University. Eight‐week‐old male BALB/c mice weighing 20–22 g were used in this study. For adriamycin‐induced proteinuria, mice received one intravenous injection of adriamycin (ADR, Sigma–Aldrich, 10 mg/kg body weight). For adoptive transfer of MDSCs, bone marrow (BM)‐derived MDSCs were prepared as previously described,^58^ and 5 × 10^6^ induced BM‐MDSCs were injected intravenously every 4 days. For dexamethasone treatment, 10 mg/kg dexamethasone (DEX) was injected intraperitoneally every day.

### 4.3 MDSC sorting and flow cytometry analysis

Monocytic and granulocytic MDSCs were sorted using a Myeloid‐Derived Suppressor Cell Isolation Kit, mouse (Miltenyi Biotec, 130‐094‐538) according to the manufacturers' protocols. Flow cytometry was performed on a Becton Dickinson FACS Calibur using CellQuest software (Becton Dickinson, Heidelberg, Germany).

### 4.4 Western blotting

Cells were lysed in buffer containing 1% Triton X‐100, a protease inhibitor cocktail and PMSF. To maintain protein phosphorylation, sodium vanadate and sodium fluoride were added when extracting the protein. Then, rabbit anti‐mouse ILT4, STAT6, pSTAT6 and GAPDH antibodies (Cell Signalling Technology, Danvers, MA) were used in the following SDS–PAGE. Binding of the antibodies was visualized using anti‐rabbit IgG‐HRP (Cell Signalling Technology, Danvers, MA) followed by ECL detection reagent (Amersham, U.K.).

### 4.5 Immunofluorescence and histochemistry

Renal sections were fixed with 4% paraformaldehyde, blocked in PBS containing 10% FBS, and then incubated with primary antibodies overnight at 4°C. Anti‐α‐SMA antibodies (Proteintech, 14,395‐1‐AP) were used as primary antibodies. FITC‐conjugated secondary antibodies were used as secondary antibodies. Then, the sections were mounted in Prolong™ Diamond Antifade Mountant (Life Technologies, P36961). Confocal images were taken using a confocal laser scanning microscope (FV3000, Olympus, USA). Paraffin sections were dewaxed, rehydrated and blocked in 3% H_2_O_2_ in 70% methanol followed by incubation for 20 min at 65°C in AP assay buffer (100 mM Tris, pH 9.5 containing 100 mM NaCl and 5 mM MgCl_2_). Coronal sections of renal tissue were stained with periodic acid‐Schiff (PAS), Masson's trichrome and Sirius Red Staining.

### 4.6 Measurement of serum cytokine levels

Whole blood was collected without anticoagulant, and the serum was isolated by centrifugation. Levels of IL‐10 and TGF‐β were determined using ELISA kits (R&D). The absorbance was measured using wavelength correction (A450 nm) with a microplate reader (Bio‐Rad).

### 4.7 Detection of albumin and creatinine

Serum/urinary creatinine and urinary albumin were measured using creatinine assay (Sigma–Aldrich) kits and mouse albumin ELISA kits (Bethyl Laboratories) according to the manufacturers' protocols.

### 4.8 T‐cell proliferation assays

For the MDSC inhibition of T‐cell proliferation assay, splenocytes were first separated with lymphocyte separation medium. Lymphocytes were labelled with CFSE according to the manufacturer's instructions (Invitrogen). CFSE‐labelled lymphocytes were stimulated with anti‐CD3 and anti‐CD28 antibodies (Invitrogen, Carlsbad, CA), and lymphocytes were cocultured at a 4:1 ratio with BM‐MDSCs in 96‐well flat bottom plates. T‐cell proliferation was analysed by flow cytometry on Day 4.

### 4.9 Quantitative real‐time PCR

Total RNA was extracted using TRIzol Reagent (Invitrogen). For mRNA quantification, total RNA was reverse transcribed to cDNA using oligo(dT). Real‐time qPCR was performed using SYBR Green normalized to GAPDH. The following PCR primers were used: TGF‐β1 forward, TGATACGCCTGAGTGGCTGTCT; TGF‐β1 reverse, CACAAGAGCAGTGAGCGCTGAA; IL‐10 forward, CGGGAAGACAATAACTGCACCC; IL‐10 reverse, CGGTTAGCAGTATGTTGTCCAGC; α‐SMA forward, ACCATCGGCAATGAGCGTTTCC; α‐SMA reverse, GCTGTTGTAGGTGGTCTCATGG; GAPDH forward, CATCACTGCCACCCAGAAGACTG; and GAPDH reverse, ATGCCAGTGAGCTTCCCGTTCAG.

### 4.10 Statistical analysis

Data are expressed as the mean ± SD. Statistical analysis was performed using Student's *t* test to assess differences between the different study groups. *p* values < 0.05 (*), <0.01 (**), <0.001 (***) and <0.0001 (****) were considered statistically significant.

## AUTHOR CONTRIBUTIONS

**Xiaowen Gu:** Data curation (equal); formal analysis (equal); investigation (equal); methodology (equal); resources (equal); software (equal); supervision (equal); validation (equal); visualization (equal); writing – original draft (lead); writing – review and editing (lead). **Lianmei Zhang:** Conceptualization (equal); data curation (equal); formal analysis (equal); investigation (equal); methodology (equal); software (equal); validation (equal); writing – original draft (equal); writing – review and editing (equal). **Min Sun:** Data curation (supporting); formal analysis (supporting); funding acquisition (supporting); investigation (supporting); methodology (supporting); resources (supporting); software (supporting); supervision (supporting); validation (supporting); visualization (supporting). **Ying Zhou:** Investigation (supporting); methodology (supporting); resources (supporting); software (supporting); supervision (supporting); validation (supporting); visualization (supporting). **Jinling Ji:** Data curation (supporting); formal analysis (supporting); investigation (supporting); methodology (supporting); resources (supporting); software (supporting); validation (supporting); visualization (supporting). **Yunfang Xu:** Data curation (supporting); formal analysis (supporting); investigation (supporting); methodology (supporting); resources (supporting); supervision (supporting); validation (supporting); visualization (supporting); writing – review and editing (supporting). **Jianguo You:** Conceptualization (lead); data curation (supporting); formal analysis (supporting); funding acquisition (lead); investigation (supporting); methodology (supporting); project administration (lead); resources (supporting); supervision (supporting); validation (supporting); visualization (supporting); writing – review and editing (supporting). **Zhikui Deng:** Conceptualization (lead); formal analysis (lead); funding acquisition (equal); methodology (equal); project administration (lead); resources (lead); supervision (lead); validation (lead); visualization (lead); writing – original draft (equal); writing – review and editing (equal).

## CONFLICT OF INTEREST STATEMENT

The authors declare no conflicts of interest.

## Data Availability

The data that support the findings of this study are available from the corresponding author, upon reasonable request.
